# A sensitive assay for measuring whole-blood responses to type I IFNs

**DOI:** 10.1073/pnas.2402983121

**Published:** 2024-09-23

**Authors:** Adrian Gervais, Corentin Le Floc’h, Tom Le Voyer, Lucy Bizien, Jonathan Bohlen, Fatih Celmeli, Fahd Al Qureshah, Cécile Masson, Jérémie Rosain, Marwa Chbihi, Romain Lévy, Riccardo Castagnoli, Anya Rothenbuhler, Emmanuelle Jouanguy, Qian Zhang, Shen-Ying Zhang, Vivien Béziat, Jacinta Bustamante, Anne Puel, Paul Bastard, Jean-Laurent Casanova

**Affiliations:** ^a^Laboratory of Human Genetics of Infectious Diseases, Necker Branch, Institut National de la Santé et de la Recherche Médicale U1163, Necker Hospital for Sick Children, Paris 75015, France; ^b^Paris Cité University, Imagine Institute, Paris 75015, France; ^c^St. Giles Laboratory of Human Genetics of Infectious Diseases, Rockefeller Branch, Rockefeller University, New York, NY 10065; ^d^Clinical Immunology Department, Assistance Publique Hôpitaux de Paris, Saint-Louis Hospital, Paris 75010, France; ^e^Division of Pediatric Allergy and Immunology, Antalya Education and Research Hospital, University of Medical Science, Antalya 07100, Türkiye; ^f^Bioinformatics Core Facility, Université Paris Cité-Structure Fédérative de Recherche Necker, INSERM US24/CNRS UMS3633, Paris 75015, France; ^g^Study Center for Primary Immunodeficiencies, Necker Hospital for Sick Children, Assistance Publique-Hôpitaux de Paris, Paris 75015, France; ^h^Pediatric Hematology-Immunology and Rheumatology Unit, Necker Hospital for Sick Children, Assistance Publique-Hôpitaux de Paris, Paris 75015, France; ^i^Pediatric Unit, Department of Clinical, Surgical, Diagnostic, and Pediatric Sciences, University of Pavia, Pavia 27100, Italy; ^j^Pediatric Clinic, Fondazione Istituto di ricovero e cura a carattere scientifico (IRCCS) Policlinico San Matteo, Pavia 27100, Italy; ^k^Endocrinology and Diabetes for children, Reference Center for rare diseases of calcium and phosphate metabolism, OSCAR network, Platform of expertise for rare diseases of Paris Saclay Hospital, Bicêtre Paris Saclay Hospital, Le Kremlin-Bicêtre 94270, France; ^l^HHMI, New York, NY 10065; ^m^Department of Pediatrics, Necker Hospital for Sick Children, Paris 75015, France

**Keywords:** auto-antibodies, type I interferons, diagnostic test, IP-10, ELISA

## Abstract

Human inborn errors of the type I IFN response pathway and auto-Abs neutralizing IFN-α, -β, and/or -ω can underlie severe viral illnesses. We report a simple assay for the detection of both types of condition. We stimulate whole blood from healthy individuals and patients with either inborn errors of type I IFN immunity or auto-Abs against type I IFNs with glycosylated human IFN-α2, -β, or -ω. As controls, we add a monoclonal antibody (mAb) blocking the type I IFN receptors and stimulate blood with IFN-γ (type II IFN). Of the molecules we test, IP-10 (encoded by the interferon-stimulated gene (ISG) *CXCL10*) is the molecule most strongly induced by type I and type II IFNs in the whole blood of healthy donors in an ELISA-like assay. In patients with inherited IFNAR1, IFNAR2, TYK2, or IRF9 deficiency, IP-10 is induced only by IFN-γ, whereas, in those with auto-Abs neutralizing specific type I IFNs, IP-10 is also induced by the type I IFNs not neutralized by the auto-Abs. The measurement of type I and type II IFN-dependent IP-10 induction therefore constitutes a simple procedure for detecting rare inborn errors of the type I IFN response pathway and more common auto-Abs neutralizing type I IFNs.

## Significance

Human inborn errors of the type I IFN pathway and auto-Abs neutralizing type I IFNs can underlie various life-threatening viral diseases, including viral pneumonias and encephalitis. Inborn errors of the type I IFN pathway are rare, but millions of people worldwide are thought to have auto-Abs neutralizing type I IFNs. There is currently no quick, easy, and affordable diagnostic test for identifying these conditions in clinical laboratories. We have filled this gap by developing a simple assay for their detection. This assay is sensitive, easy, robust, affordable, and provides results quickly, thus meeting the requirements of clinical laboratories.

Auto-Abs against type I IFNs were first described in 1981–1984, in a single patient with disseminated shingles (1–3). They may be produced due to monogenic inborn errors of tolerance to self, as in almost all patients with autosomal recessive autoimmune polyendocrine syndrome type 1 (APS-1), a disease caused by biallelic loss-of-function *AIRE* variants (4–8). Other related genetic etiologies include deleterious variants in men hemizygous for deleterious X-linked *FOXP3* variants (9) (causing immune dysregulation polyendocrinopathy enteropathy X-linked (IPEX) recessive syndrome) or women heterozygous for deleterious X-linked *IKBKG* variants (10, 11) causing incontinentia pigmenti (IP) a dominant X-linked disorder. Biallelic variants of the autosomal genes *RAG1*, *RAG2* (8, 12–14), *NIK*, and *RELB* (15), and monoallelic variants of *AIRE* (7, 16), *NFKB2* (15), *IKZF2* (17, 18), and *CTLA4* (17) can also underlie the production of these auto-Abs. These genes are expressed in thymocytes (e.g., *RAG1*, *RAG2*, *FOXP3*, *IKZF2*, *CTLA4, NFKB2, IKBKG*) and/or in thymic stromal cells (*AIRE*, *IKBKG*, *IKZF2*, *NFKB2, NIK, RELB*) and/or in T cells (*FOXP3, IKZF2, CTLA4, NFKB2, IKBKG, NIK, RELB)*, and their defects impair central T-cell tolerance. Auto-Abs against type I IFNs can also be found in patients with conditions with a less well-delineated genetic architecture, including myasthenia gravis and/or thymoma (30 to 75%), systemic lupus erythematosus (SLE) (∼10%), and in patients treated with type I IFN (19–25). In the general population, they are found in <0.5% (auto-Abs neutralizing high concentrations of type I IFNs) and <2% (auto-Abs neutralizing low concentrations of type I IFNs) of individuals under the age of 65 y, and their prevalence increases sharply thereafter, reaching ∼4% and ∼8%, respectively, in individuals over the age of 70 y (26, 27). The underlying cause of the auto-Abs neutralizing type I IFNs remains unexplained in most cases.

Auto-Abs against type I IFNs were long thought to be clinically silent (1, 27–30). Their discovery in APS-1 patients in 2006 led to their use of their detection as a diagnostic marker for this condition (4–6). It has been suggested that they influence the course of diabetes in APS-1 patients (6), and the severity of disease in SLE patients (21, 22, 31, 32). However, it was not until the COVID-19 pandemic that their pathogenic role in viral diseases was widely accepted. Most unvaccinated SARS-CoV-2-infected APS-1 patients were hospitalized for hypoxemic COVID-19 pneumonia (33–35). Moreover, ~15% of cases of critical COVID-19 pneumonia were found to be due to preexisting auto-Abs neutralizing type I IFNs (10, 22, 26, 36, 37). These findings were replicated worldwide (31, 34, 38–70). Auto-Abs neutralizing type I IFNs also underlie ∼5% of cases of critical influenza pneumonia (71), ∼25% of hospitalizations for Middle East respiratory syndrome (MERS) pneumonia (27, 30, 72), ∼30 to 40% of severe adverse reactions to the live attenuated virus vaccine against yellow fever virus (YFV-17D) (73, 74) and, strikingly, ∼40% of cases of West Nile virus encephalitis (75). These auto-Abs have a major clinical impact, with odds ratios (OR) for severe disease in carriers increasing with the concentration and number of type I IFNs neutralized, typically reaching values greater than 10, and often greater than 100 (26, 29, 75). These auto-Abs are present before viral infection and are causal for severe disease (29, 37).

Current procedures for detecting auto-Abs neutralizing type I IFNs are based on in vitro cell-based assays. They usually involve assessments of STAT1 phosphorylation (p-STAT1) by flow cytometry after the stimulation of peripheral blood mononuclear cells (PBMCs) with type I IFNs in the presence of plasma or serum from the patient or control (8–10, 76). More sensitive luciferase reporter cell-based assays have also recently been developed (26). In the STAT1 phosphorylation assays, the blocking activity of auto-Abs is evaluated by assessing STAT1 phosphorylation in healthy control PBMCs stimulated with type I IFNs in the presence of 10% serum or plasma from the patient or a healthy control (8–10, 76). In the luciferase assays, HEK293T cells are transfected with a plasmid containing the firefly luciferase gene under the control of a promotor inducible by type I IFNs due to the presence of five interferon-sensitive response element (ISRE) repeats, and a plasmid encoding the *Renilla* luciferase (as a control for transfection) (26). The cells are then stimulated with type I IFNs in the presence of 10% plasma or serum from a patient or a healthy control. If this plasma or serum contains neutralizing auto-Abs against type I IFNs, these cytokines are neutralized and, thus, unable to signal through their receptors, resulting in low levels of firefly luciferase activity. By contrast, in the absence of such antibodies, the type I IFNs can induce high levels of firefly luciferase activity.

These assays are robust and sensitive, but time-consuming, labor-intensive, and expensive. The flow cytometry assays take 1 to 2 d, and the luciferase assay, 4 d. Moreover, the flow cytometry-based assays have a limited throughput, preventing their use to screen large numbers of individuals. Both assays are also costly, at an estimated US$6 to 10 for the testing of a single sample. These limitations account for the restriction of their use to only a small number of laboratories worldwide, typically research laboratories in the wealthiest countries. Furthermore, these assays use 1:10 dilutions of plasma, and this dilution may result in an underestimation of the prevalence of auto-Abs against type I IFNs. Finally, methods for auto-Ab detection based on antibody structure (ELISA) rather than neutralizing function are simpler and cheaper, but nevertheless have drawbacks, as neutralizing auto-Abs can escape ELISA detection, and the auto-Abs detected by ELISA may be nonneutralizing (26, 35, 75, 77). Indeed, the correlation between structural and functional detections of these auto-Abs depends on many factors, only some of which are known (78–80). The detection of neutralizing auto-Abs is globally important, in terms of public health, as there are probably at least 100 million carriers and the spectrum of known clinical consequences of these auto-Abs is continually expanding. We therefore set out to develop a simple, quick, affordable method for detecting auto-Abs neutralizing type I IFNs that would be easy to implement in clinical practice and in clinical laboratories worldwide.

## Results

### IP-10 Is the Most Strongly Induced by Type I IFNs in Blood of All the Molecules Tested.

**Fig. 1.**
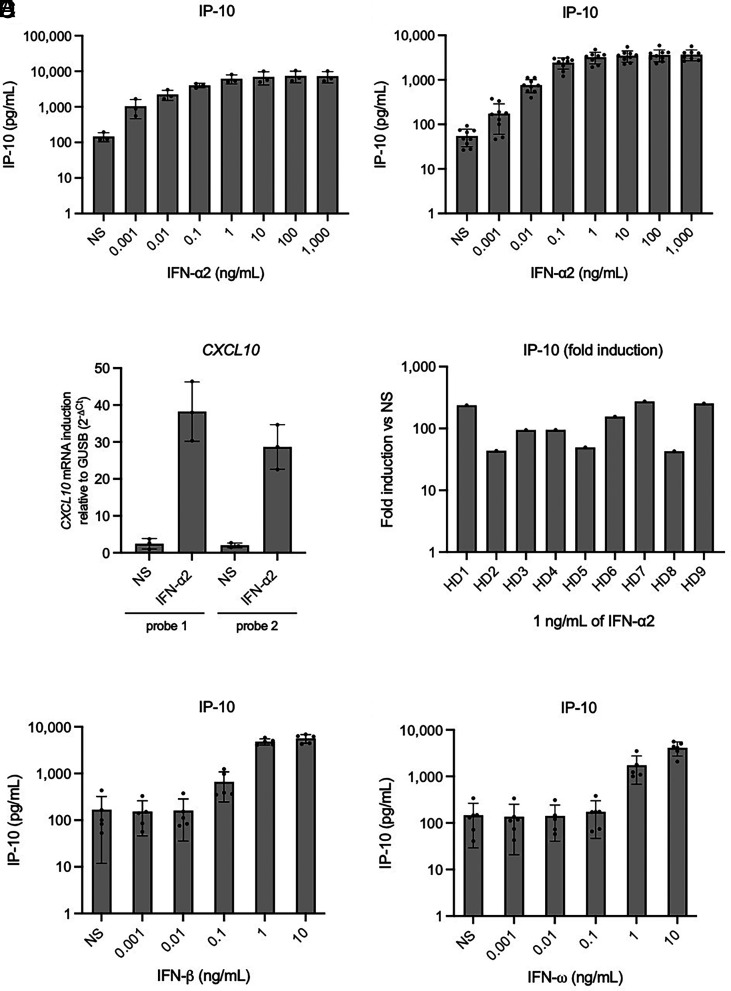
IP-10 (*CXCL10*) induction after the stimulation of whole blood from healthy donors with type I IFNs. (*A* and *B*) IP-10 induction, assessed with plasma supernatants after the stimulation of whole blood with various concentrations of glycosylated IFN-α2 for 16 h, as measured by LEGENDplex™. Blood samples were collected 8 to 24 h before stimulation. Three (*A*) or nine (*B*) healthy donors were tested once for each set of conditions. (*C*) *CXLC10* mRNA induction after the stimulation of PBMCs from three healthy donors with 1 ng/mL glycosylated IFN-α2 for 6 h, as measured by RT-qPCR. (*D*) Fold-induction of IP-10 after the stimulation of whole blood with 1 ng/mL glycosylated IFN-α2 for 16 h, as measured by LEGENDplex™. Nine healthy donors were tested once each. Blood samples were collected 8 to 24 h before stimulation. (*E* and *F*) IP-10 induction, assessed with plasma supernatants after the stimulation of whole blood with various concentrations of glycosylated IFN-β (*E*) or glycosylated IFN-ω (*F*) for 16 h, as measured by LEGENDplex™. Blood samples were collected 8 to 24 h before stimulation. Five healthy donors were tested once for each set of conditions.

We stimulated whole blood (collected in lithium heparin tubes 4 to 48 h before stimulation and kept at room temperature with gentle shaking) from three healthy donors with various concentrations (1 pg/mL to 1 µg/mL) of glycosylated recombinant human IFN-α2, or left it unstimulated (nonstimulated, NS). After 16 h, we assessed the production of a panel of 25 potentially relevant cytokines and chemokines (IL-1β, IL-6, TNF, IP-10, IFN-λ1, IL-8, IL-12p70, IFN-λ2/3, GM-CSF, IL-10, MCP-1, IL-17A, IL-18, IL-23, IL-33, Eotaxin, TARC, RANTES, MIP-1α, MIG, ENA-78, MIP-3α, GROα, I-TAC, and MIP-1β) in LEGENDplex™ multiplex assays (an ELISA-like assay) on the supernatant. IP-10 (encoded by *CXCL10*) had the highest absolute induction levels of all the proteins tested, for all three controls (with a mean fold-induction over nonstimulation of 42-fold for stimulation with 1 ng/mL), and with detectable but low levels at baseline (Fig. 1*A*). IL-6 had the second-highest absolute induction levels, but in a much lower extent (fourfold), also varying considerably between the individuals tested (*SI Appendix*, Fig. S1*A*), while the remaining molecules tested were poorly induced. At the mRNA level, RT-qPCR revealed a strong induction of *CXCL10* expression in the fresh PBMCs of the three healthy donors tested after 6 h of stimulation with 1 ng/mL IFN-α2 (Fig. 1*B*). A study of nine additional healthy donors confirmed the consistent production of IP-10 in response to type I IFN stimulation, with detection by LEGENDplex™ (Fig. 1*C*). With 1 ng/mL IFN-α2, a 30- to 100-fold induction (mean: 70-fold, SD: 40-fold) was observed for IP-10 protein levels, and a 10- to 80-fold induction (mean: 30-fold, SD: 20) was observed for *CXCL10* mRNA levels (Fig. 1*D* and *SI Appendix*, Fig. S1*B*). Finally, we measured IP-10 induction after stimulation with the other two major type I IFNs: IFN-β and IFN-ω. Stimulation with 1 or 10 ng/mL glycosylated IFN-β induced high levels of IP-10 (>4,000 pg/mL), whereas lower concentrations of IFN-β resulted in little or no induction (Fig. 1*E*). Glycosylated IFN-ω induced IP-10 less strongly than IFN-α2 and IFN-β, but the resulting IP-10 levels were high enough for consistent detection when 1 ng/mL IFN was used for stimulation (Fig. 1*F*). Overall, we identified IP-10 as a protein strongly induced by type I IFNs and easily detectable by an ELISA-like assay on blood. The use of an IFN concentration of 1 ng/mL yielded a robust response for all three type I IFNs tested and a sensitivity similar to that of the previously described classical luciferase-based neutralization assay (26).

### Genome-Wide Expression Analysis Confirms the Suitability of IP-10 as a Candidate Target.

**Fig. 2.**
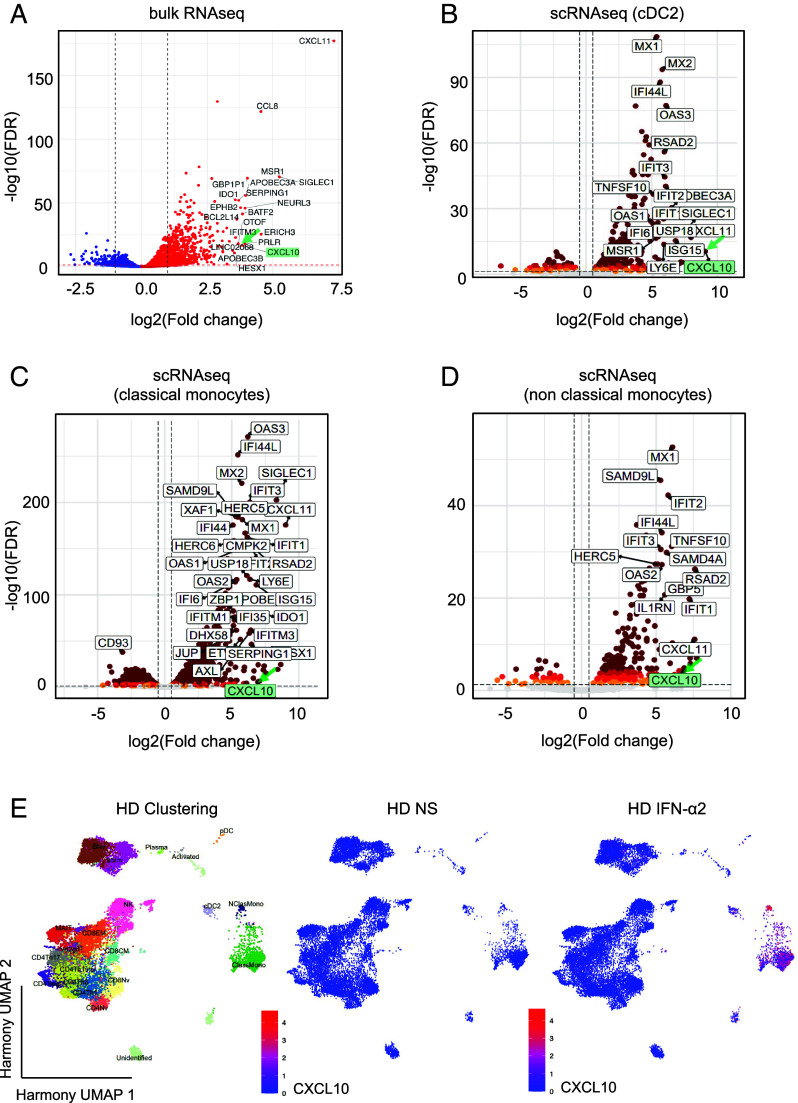
Whole-transcriptome analysis after PBMC stimulation with IFN-α2. (*A*) Volcano plot analysis of bulk RNAseq performed on total mRNA extracted from the PBMCs of three healthy donors after stimulation with 1 ng/mL glycosylated IFN-α2 for 6 h. The labeled genes are the top 20 genes displaying the highest levels of induction relative to nonstimulated conditions. (*B*–*D*) Volcano plot analysis showing the transcripts induced in cDC2 cells (*B*), classical monocytes (*C*), and nonclassical monocytes (*D*), as determined by scRNAseq (Parse Bioscience). PBMCs of three healthy donors were stimulated with 1 ng/mL glycosylated IFN-α2 for 6 h and whole mRNA was extracted. (*E*) Single-cell transcriptom analysis. PBMCs from three HD were analyzed after IFN-α2 stimulation vs unstimulated. Clustering analysis. After batch correction with Harmony, celltypes were identified manually. Feature plots show the induction of *CXCL10* (encoding IP-10).

We then used an unbiased genome-wide approach to determine whether IP-10 was a strong candidate. We stimulated fresh PMBCs from three healthy donors with 1 ng/mL IFN-α2 for 6 h and performed bulk RNAseq. We found that *CXCL10* was among the top 20 most strongly induced transcripts (Fig. 2*A*). We corroborated these results by performing single-cell (sc)RNAseq analysis on the same samples, revealing high levels of *CXCL10* induction by IFN-α2 in type 2 conventional dendritic cells (cDC2), classical and nonclassical monocytes, naive and memory B cells, memory CD8, CD4 Th2 cells, and CD4 Th17 T cells and NK cells (Fig. 2 *B*–*E* and Table 1). We also determined whether the proteins corresponding to the other 19 of the top 20 transcripts were expressed at the cell surface or secreted, and whether mAbs were commercially available for their detection (Table 2). We measured the induction of these potential targets by ELISA after 16 h of stimulation with type I IFNs. We found that I-TAC (encoded by *CXCL11*, the most strongly induced gene identified in the RNAseq experiment) was poorly detectable, with high background levels for protein detection (*SI Appendix*, Fig. S2*A*). CD169 (encoded by *SIGLEC1*) induction by IFN-α2 or IFN-β gave only a weak signal (maximum of 750 pg/mL and 1,000 pg/mL, respectively), with a relatively high background (250 pg/mL in the absence of stimulation) (*SI Appendix*, Fig. S2 *B* and *C*). Stimulation with IFN-ω resulted in generally higher levels of CD169 production (2,000 pg/mL) (*SI Appendix*, Fig. S2*D*). Whole-blood stimulation with 1 ng/mL type I IFN resulted in no more than an eight-fold induction of CD169, much weaker than the induction observed for IP-10, and CD169 was therefore not considered a strong candidate for the assay in the conditions tested. Finally, we tested MCP-2 (encoded by *CCL8*) as the fourth and final secreted protein displaying high levels of induction at the mRNA level according to RNAseq results (*SI Appendix*, Fig. S2 *E*-*G*). We found that MCP-2 was less strongly induced by 1 ng/mL IFN-α2 (median: 204 pg/mL), IFN-β (median: 834 pg/mL), and IFN-ω (median: 54 pg/mL) than IP-10, as determined by LEGENDplex™. Overall, IP-10 remained the most robust candidate among the targets most strongly induced in the conditions tested.

**Table 1.** *CXCL10* expression in different cell subpopulations

Cell type	log2FC	*P* value	Adjusted *P* value
cDC2	9.08043133	3.93E-13	3.41E-11
ClassMono	7.07152808	1.81E-08	2.63E-07
NClasMono	6.88990241	1.12E-06	4.19E-05
BNv	4.93703754	1.58E-43	8.21E-42
CD8CM	4.62740916	1.29E-06	4.49E-05
BMm	4.41136895	4.40E-43	3.41E-41
CD4Th2	3.16694567	1.62E-10	3.68E-09
CD4Th17	2.81563776	3.97E-06	6.48E-05
NK	2.35180089	7.48E-06	9.97E-05
CD4Treg	2.58354929	1.30E-02	1.05E-01
CD4Th1	1.54400230	2.85E-02	1.10E-01
MAIT	1.40797691	4.32E-02	2.27E-01
CD8Nv	1.10810034	1.02E-01	2.62E-01

PBMCs from three healthy donors were stimulated with 1 ng/mL IFN-α2 for 6 h, and scRNAseq was performed (Parse Bioscience). cDC2: type 2 conventional dendritic cells; ClassMono: classical monocytes; NClasMono: nonclassical monocytes; BNv: naive B cells; CD8CM: CD8^+^ central memory T cells; BMm: memory B cells; CD4Th2: CD4^+^ T helper 2 cells; CD4Th17: CD4^+^ T helper 17 cells; NK: natural killer cells; CD4Treg: CD4^+^ T regulator cells; CD4Th1: CD4^+^ T helper 1 cells; MAIT: mucosal-associated invariant T cells; CD8Nv: CD8^+^ naive T cells.

**Table 2.** Top 20 genes induced by the stimulation of PBMCs from three healthy donors with 1 ng/mL IFN-α2 for 6 h, as determined by RNAseq

Gene	Mode of expression	Expression in immune cells at RNA level	Detected in blood immunoassays (Human Protein Atlas, HPA)	Commercial mAb available
*CXCL10*	Secreted	Monocytes	Yes	Yes
*CXCL11*	Secreted	Monocytes	Yes	Yes
*CCL8*	Secreted	Monocytes	Yes	Yes
*SIGLEC1*	Membrane, intracellular, secreted	Monocytes	No	Yes
*MSR1*	Membrane, intracellular	Monocytes	No	—
*EPHB2*	Membrane, intracellular	Monocytes	No	—
*OTOF*	Membrane, intracellular	Monocytes, T	No	—
*IFITM3*	Membrane, intracellular	Monocytes, neutrophils	No	—
*PRLR*	Membrane, intracellular	Monocytes, neutrophils	No	—
*APOBEC3A*	Intracellular	Monocytes	No	—
*SERPING1*	Intracellular	Dendritic, neutrophils	No	—
*IDO1*	Intracellular	Eosinophils	No	—
*NEURL3*	Intracellular	Not detected	No	—
*BCL2L14*	Intracellular	T	No	—
*BATF2*	Intracellular	Monocytes, neutrophils	No	—
*ERICH3*	Intracellular	Not detected	No	—
*APOBEC3B*	Intracellular	Monocytes, memory B	No	—
*HESX1*	Intracellular	Monocytes, neutrophils	No	—
*GBP1P1*	Pseudogene	—	—	—
*LINC02068*	RNA gene	—	—	—

For each gene, we indicate the mode of expression of the corresponding protein, the immune cells in which its RNA is found according to public databases (Human Protein Atlas), whether the corresponding protein is detected in blood immunoassays (Human Protein Atlas), and whether monoclonal antibodies are commercially available.

### IP-10 Is Also Induced After Whole-Blood Stimulation with IFN-γ but Not with IFN-ε and IFN-κ.

**Fig. 3.**
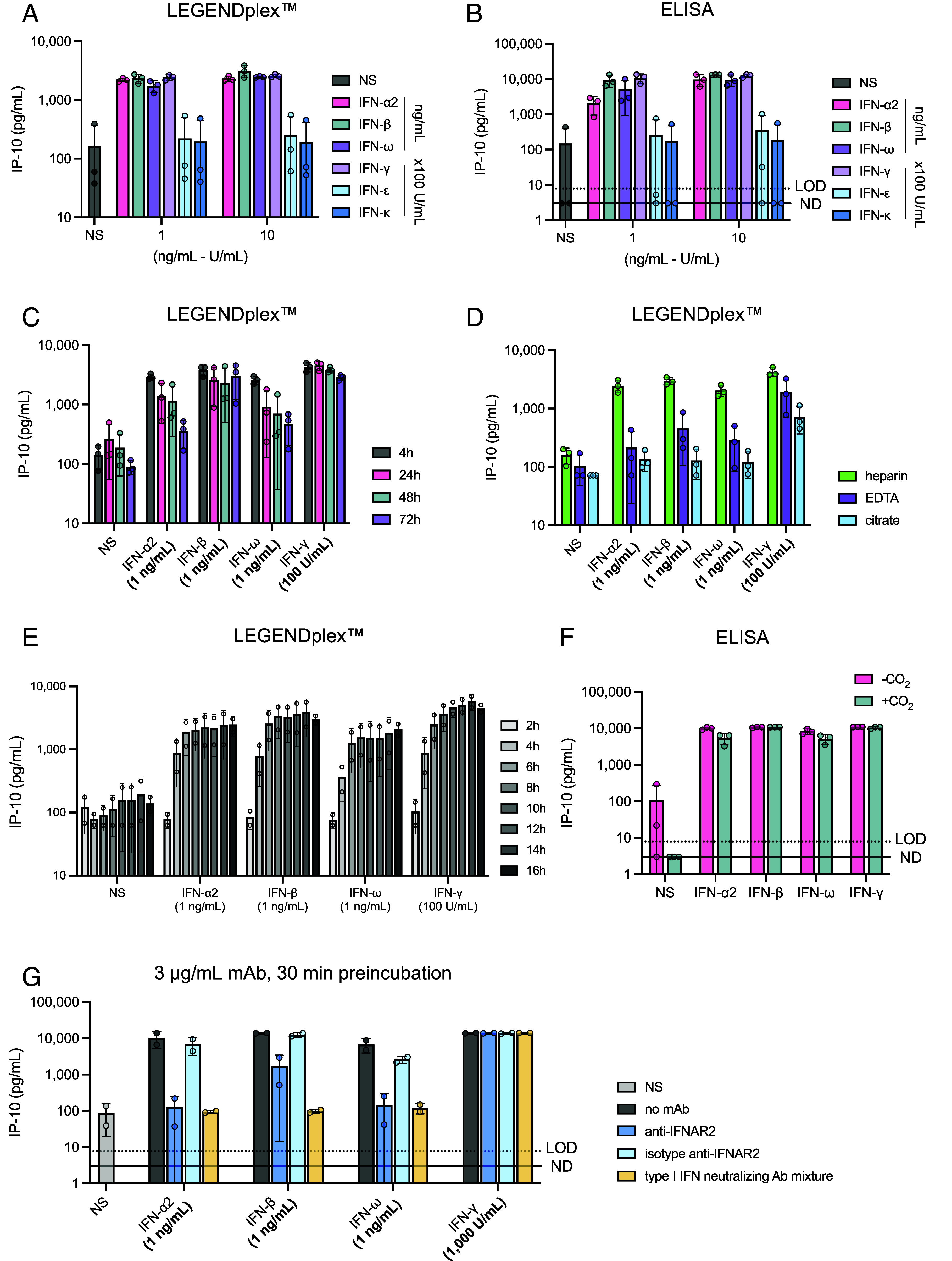
Evaluation of technical parameters and assessment of the activity of other cytokines (human IFN-ε, IFN-κ, and IFN-γ) on IP-10 induction. (*A*) IP-10 induction after stimulation with 100 U/mL or 1,000 U/mL recombinant human IFN-ε, IFN-κ, or IFN-γ for 16 h. Three healthy donors were tested for each set of conditions. IP-10 levels were then assessed in plasma supernatants by LEGENDplex™. (*B*) IP-10 induction after stimulation with 100 U/mL or 1,000 U/mL recombinant human IFN-ε, IFN-κ, or IFN-γ for 16 h. Three healthy donors were tested for each set of conditions. IP-10 levels were then assessed in plasma supernatants by ELISA. (*C*) Effect of time between collection and stimulation on IP-10 induction. IP-10 levels were assessed in plasma supernatants by LEGENDplex™ after the stimulation of whole blood with 1 ng/mL IFN-α2, -β, -ω, or -γ for 16 h. Three healthy donors were tested for each set of conditions. (*D*) Effect of the matrix on which blood is collected. Lithium heparin, EDTA, and citrate tubes were used to collect blood from three healthy donors. Whole-blood samples were stimulated with 1 ng/mL IFN-α2, -β, -ω, or -γ for 16 h. IP-10 levels were then assessed in plasma supernatants by LEGENDplex™. (*E*) Kinetics of IP-10 induction (effect of stimulation time). Whole blood from two healthy donors was stimulated with the indicated concentrations of IFNs for 2, 4, 6, 8, 10, 12, 14, and 16 h. IP-10 levels were then assessed in plasma supernatants by LEGENDplex™. (*F*) Effect of the presence or absence of 5% CO_2_ on IP-10 production after the stimulation of whole blood from three healthy donors with 1 ng/mL IFN-α2, -β, -ω, or -γ for 16 h. −CO_2_: stimulation at 37 °C without CO_2_ supplementation, +CO_2_: stimulation at 37 °C with 5% CO_2_ supplementation. (*G*) IP-10 induction after the stimulation of whole blood from two HDs with type I IFNs and with preincubation with 3 µg/mL anti-IFNAR2 neutralizing mAb, isotype antibody, or a cocktail of antibodies neutralizing all type I IFNs (dilution 1:50). The mAbs were incubated with whole blood for 30 min before stimulation with IFNs.

Human IFN-ε and IFN-κ are restricted to the female reproductive tract and the skin, respectively, unlike the other type I IFNs, which are broadly expressed (81–83). Efforts to detect auto-Abs neutralizing IFN-ε or IFN-κ have, therefore, been limited, as such antibodies would be expected to have a narrower biological and medical relevance than those against IFN-α, IFN-β, or IFN-ω. Moreover, these IFNs with low affinity for their receptor are unable to induce detectable STAT1 phosphorylation and firefly luciferase activity in the widely used neutralization assays, even at high concentrations, precluding the detection of auto-Abs targeting them in these assays. This poor detection probably results from their affinity for the IFNAR1-IFNAR2 heterodimer being more than three orders of magnitude lower than that of the other type I IFNs (84). We nevertheless evaluated the ability of these IFNs to induce IP-10 following the stimulation of fresh, whole-blood samples, in the conditions described above. We also assessed the activity of IFN-γ in this assay to serve as a control for blood stimulation, as IP-10 has been reported to be type II IFN-inducible (85). We thought of using an IFN-α from another primate species as a control but we found that cynomolgus monkey IFN-α2, which is 92% identical to human IFN-α2, stimulated human cells but was also neutralized by human auto-Abs against IFN-α2. Stimulation with 10 ng/mL IFN-ε or IFN-κ (the highest concentration used) led to the detection of IP-10 at levels similar to those observed in the absence of stimulation, suggesting that these IFNs were unable to induce IP-10, at least in these conditions of stimulation and detection in cells (Fig. 3*A*). By contrast, stimulation with 100 U/mL or 1,000 U/mL recombinant IFN-γ (IFN-γ1b, unglycosylated) led to the production of almost 3,000 pg/mL IP-10, a level similar to that observed with 1 to 10 ng/mL IFN-α2, IFN-β, or IFN-ω. This finding is consistent with IP-10 being inducible by IFN-γ and suggests that our test could also be used to detect auto-Abs neutralizing IFN-γ or to diagnose inborn errors of IFN-γ-dependent immunity (77, 78). IFN-γ can, therefore, be used at least as a control for tests on patients with inborn errors of the type I IFN-dependent and type II IFN-independent response pathways, and patients with auto-Abs against type I but not type II IFNs. It should be noted that the blood samples used here were collected more than 24 h before the assay, which may have decreased the amount of IP-10 detected. A classical ELISA was performed on these plasma supernatants and yielded similar results, with slightly higher sensitivity (Fig. 3*B*). Thus, IP-10 can be induced by the three major human type I IFNs, but not by the tissue-restricted IFN-ε and IFN-κ, and it is also induced by type II IFN. IP-10 is, therefore, a suitable target molecule for the detection of auto-Abs neutralizing the corresponding type I or type II IFNs. Moreover, IFN-γ can be used as a control in screens for auto-Abs neutralizing type I IFNs only, and vice versa.

### Assessment of Experimental Conditions Likely to Affect IP-10 Production.

We then investigated technical parameters that might modify IP-10 induction in the assay, such as the time between blood sampling and stimulation, and the matrix used for blood sampling (containing clot activators or anticoagulants). We tested the blood of three healthy donors sampled 4, 24, 48, and 72 h before stimulation with 1 ng/mL IFN. The strongest IP-10 response was obtained following the stimulation of blood samples obtained 4 h before the assay (3,000 to 4,000 pg/mL) (Fig. 3*C*). Collecting the blood sample 24 h before stimulation almost halved the amount of IP-10 detected in the assay, although the response to IFN-γ remained strong. IP-10 production after stimulation with type I IFNs was similar for blood samples collected 24 or 48 h before the assay but was much lower for blood samples collected 72 h before the assay (Fig. 3*C*). We then compared the IP-10 response to stimulation with IFN between blood samples collected in tubes containing lithium heparin, ethylenediamine tetraacetic acid (EDTA), or citrate. Blood collected in lithium heparin and stimulated with 1 ng/mL type I IFN produced 2,000 to 3,000 pg/mL IP-10, but IP-10 levels were much lower for blood samples collected in EDTA (<600 pg/mL) and almost no IP-10 was detected for blood samples collected in citrate (Fig. 3*D*). We then studied the kinetics of IP-10 production following IFN stimulation. We stimulated blood samples collected in lithium heparin from two healthy donors with 1 ng/mL type I IFN and 100 U/mL IFN-γ, 16 h after sampling. Plasma supernatants were collected 2, 4, 6, 8, 10, 12, 14, and 16 h poststimulation for the assessment of IP-10 production. IP-10 was detectable as early as 4 h after stimulation, and relatively high levels were detected after 6 h of stimulation (Fig. 3*E*). Beyond this timepoint, IP-10 levels continued to increase, slowly but steadily, to reach a plateau after 14 to 16 h of stimulation. Finally, we compared IP-10 responses following the stimulation with IFN of blood incubated with and without 5% CO_2_, as clinical laboratories do not use incubators with CO_2_-enriched atmospheres. We found no difference in IP-10 induction between these conditions (Fig. 3*F*). Finally, we showed that mAbs blocking IFNAR2 or a cocktail of mAbs neutralizing all type I IFNs prevented or strongly impaired the induction of IP-10 by any type I IFN (Fig. 3*G*). This observation provided an important control for diagnostic purposes for patients with suspected auto-Abs against type I IFNs or genetic deficiencies of the type I IFN response pathway. Overall, these data suggest that, for optimal results, blood should be i) stimulated soon after sampling, preferably within 24 h, ii) imperatively collected in lithium heparin-containing tubes, and iii) stimulated for at least 6 h and, ideally, between 14 and 16 h.

### IP-10 Is Not Induced by Type I IFNs in Patients with Auto-Abs Neutralizing Type I IFNs.

**Fig. 4.**
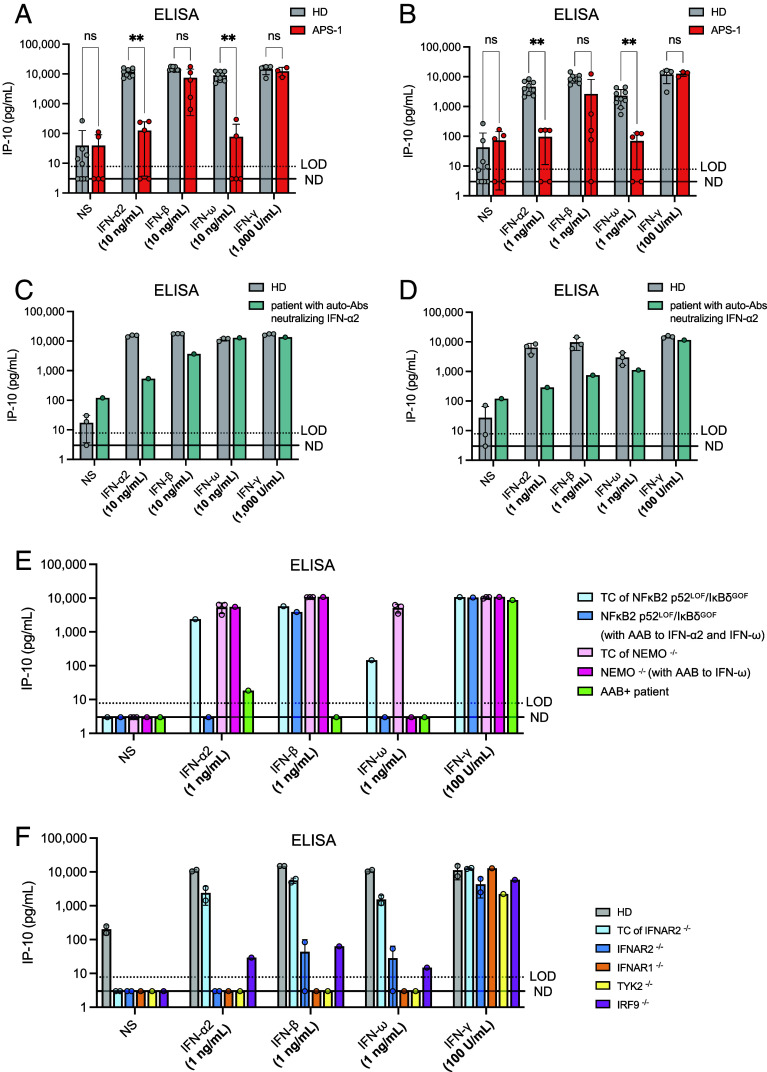
IP-10 induction after the stimulation of whole blood from patients with impaired type I IFN-dependent immunity. (*A*) IP-10 induction after the stimulation of whole blood from five APS-1 patients and nine healthy donors with 10 ng/mL glycosylated IFN-α2, IFN-β, or IFN-ω for 16 h. IP-10 levels were measured in plasma supernatants by ELISA. Whole blood from three APS-1 patients and five healthy donors was also stimulated with 1,000 U/mL IFN-γ as a control. IP-10 levels were compared between the HD and APS-1 groups by implementing nonparametric Mann–Whitney tests in GraphPad Prism. Ns: not significant; **P-value < 0.001. (*B*) IP-10 induction after the stimulation of whole blood from five APS-1 patients and nine healthy donors with 1 ng/mL glycosylated IFN-α2, IFN-β, or IFN-ω for 16 h. IP-10 levels were measured in plasma supernatants by ELISA. Whole blood from three APS-1 patients and five healthy donors was also stimulated with 100 U/mL IFN-γ as a control. Multiple Mann–Whitney tests were performed to compare the HD and APS-1 groups for each set of stimulation conditions. IP-10 levels were compared between the HD and APS-1 groups in nonparametric Mann–Whitney tests implemented in GraphPad Prism. Ns: not significant; **P-value < 0.001. (*C*) IP-10 induction in plasma supernatants after the stimulation of whole blood from a patient neutralizing low concentrations of IFN-α2 (1 ng/mL to 100 pg/mL) in a luciferase assay. Whole-blood samples from the patient and three healthy donors were stimulated with glycosylated type I (10 ng/mL) or type II (1,000 U/mL) IFNs for 16 h. IP-10 levels were then assessed in plasma supernatants by ELISA. (*D*) IP-10 induction in plasma supernatants after the stimulation of whole blood from a patient neutralizing low concentrations of IFN-α2 (1 ng/mL to 100 pg/mL) in a luciferase assay. Whole-blood samples from the patient and three healthy donors were stimulated with glycosylated type I (1 ng/mL) or type II (100 U/mL) IFNs for 16 h. IP-10 levels were then assessed in plasma supernatants by ELISA. (*E*) IP-10 induction in plasma supernatants after the stimulation of whole blood from a patient with a *NFKB2* p52^LOF^/IκBδ^GOF^ mutation, a female patient with NEMO deficiency (incontinentia pigmenti), and a patient with auto-Abs neutralizing type I IFNs with no genetic diagnosis. TC: Travel control. Whole-blood samples were stimulated with glycosylated type I (1 ng/mL) or type II (100 U/mL) IFNs for 16 h. IP-10 levels were then assessed in plasma supernatants by ELISA. (*F*) IP-10 induction in plasma samples from patients with autosomal recessive IFNAR1, IFNAR2, TYK2, or IRF9 deficiency. Whole-blood samples from the patients and three healthy donors were stimulated with glycosylated type I (1 ng/mL) or type II (100 U/mL) IFNs for 16 h. IP-10 levels were then assessed in plasma supernatants by ELISA. A TC blood sample from a healthy individual was available for one of the IFNAR2^−/−^ patients. These samples were stimulated >48 h after blood sampling. The blood of the TYK2^−/−^ patient was stimulated 24 h after blood sampling. The blood of the IRF9^−/−^ patient was stimulated 6 h after blood sampling.

We collected whole-blood samples in lithium heparin, for nine healthy individuals and five APS-1 patients. These samples were stimulated, 8 to 24 h after their collection, with 10 ng/mL or 1 ng/mL of IFN-α2, IFN-β, or IFN-ω. Plasma samples from the healthy donors were simultaneously tested negative for auto-Abs neutralizing type I IFNs, whereas plasma samples from the five APS-1 patients were previously found to neutralize 10 ng/mL IFN-α2a and IFN-ω, and one of these samples also neutralized 1 ng/mL IFN-β. Of note, these neutralization values were obtained in a luciferase-based neutralization assay in the presence of a 1:10 dilution of plasma, implying a neutralization of 10 times higher concentrations in vivo. After 16 h of stimulation, we measured IP-10 levels in the supernatant by ELISA (Fig. 4 *A* and *B*). After stimulation with 10 ng/mL IFNs, strong IP-10 induction (up to >10,000 pg/mL) was observed for the nine healthy donors tested, in all conditions of stimulation (Fig. 4*A*). By contrast, tests on the five APS-1 patients revealed an abolition of IP-10 induction after stimulation with IFN-α2 or IFN-ω, thereby demonstrating the neutralizing activity of the auto-Abs of these patients. Blood samples from five healthy donors and three APS-1 patients were also stimulated with 1,000 U/mL IFN-γ, leading to strong IP-10 induction in the samples of both patients and controls. After stimulation with 1 ng/mL IFNs, IP-10 levels remained high in blood from healthy donors, whereas IFN-α2 and IFN-ω were completely neutralized by all blood samples from APS-1 patients; the samples from three of these patients completely neutralized IFN-β and the sample from another one of these patients partially neutralized IFN-β (Fig. 4*B*). IP-10 induction was similar in patients and healthy donors after stimulation with 100 U/mL IFN-γ. We then tested blood from a patient with severe COVID-19 pneumonia and auto-Abs neutralizing low concentrations of IFN-α2 (i.e., 1 ng/mL and 100 pg/mL, as previously tested in luciferase-based neutralization assays), but without a genetic diagnosis. In the whole-blood assay, blood from this patient neutralized 10 ng/mL IFN-α2 (equivalent to 1 ng/mL IFN-α2 in the luciferase assay, due to the 1:10 dilution of the plasma). The patient’s blood sample also completely neutralized 1 ng/mL IFN-α2 (equivalent to 100 pg/mL in the luciferase assay) and, interestingly, also partially neutralized 1 ng/mL IFN-β and IFN-ω, whereas the samples from the healthy donors did not (Fig. 4 *C* and *D*). IP-10 production following IFN-γ stimulation was normal in both the patient and controls. Finally, we tested blood from a patient heterozygous for an autosomal *NFKB2* p52^LOF^/IκBδ^GOF^ allele carrying auto-Abs neutralizing 10 ng/mL IFN-α2 and IFN-ω, a woman heterozygous for an X-linked *NEMO*^LOF^ allele carrying carries auto-Abs neutralizing 10 ng/mL IFN-ω, and a patient with auto-Abs neutralizing 10 ng/mL of all type I IFNs with no genetic diagnosis. These neutralization data previously obtained in the luciferase assay were fully reproduced in our whole-blood IP-10 assay (Fig. 4*E*). Thus, IP-10 is not induced by type I IFNs in patients with auto-Abs neutralizing high or low concentrations of the corresponding type I IFNs. This assay is at least as sensitive as the luciferase assay, and perhaps even more sensitive, given that it uses whole blood rather than plasma.

### IP-10 Is Not Induced by the Activation of Whole Blood from Patients with IEI Affecting the Type I IFN Response Pathway.

We assessed the impact of a genetic deficiency of the type I IFN response pathway by stimulating fresh blood from patients with autosomal recessive, complete IFNAR1 (86–89), IFNAR2 (90, 91), TYK2 (92–94), or IRF9 (95) deficiency. Leukocytes from patients with complete TYK2 deficiency display a profound, but not complete impairment of responses to type I IFN, IL-10, IL-12, and IL-23 (92–94). Following the stimulation of whole blood from a TYK2-deficient patient or from an IFNAR1-deficient patient with 1 ng/mL IFN-α2, IFN-ω, or IFN-β, IP-10 was produced in amounts similar to those observed in the absence of stimulation, attesting to a complete lack of response; by contrast, blood from the healthy donors tested responded normally. IP-10 production after IFN-γ stimulation was normal in both patients and controls (Fig. 4*F*). The results for one of the two IFNAR2-deficient patients were identical, with a complete lack of response, whereas the other IFNAR2-deficient patient produced very small, but nevertheless detectable amounts of IP-10 after stimulation with IFN-β or IFN-ω. This was perhaps due to the affinity of these two IFNs for their receptors (IFNAR1 and IFNAR2) being higher than that of IFN-α2 for its receptor, allowing some signal transduction through IFNAR1 alone (96) (Fig. 4*F*). The levels of IP-10 produced following stimulation with IFN-γ were slightly lower for the IFNAR2-deficient patients than for the three healthy donors (but similar to that of the travel control of the IFNAR2-deficient patient) and the TYK2-deficient patient, probably because their blood samples were not stimulated until 48 and 36 h after their collection. This highlights the necessity of a (travel) control sample drawn at the same time as that of the patient, to ensure identical conditions and correct interpretation. Interestingly, the IRF9-deficient patient displayed weak but detectable IP-10 induction after stimulation with all type I IFNs, perhaps mediated by STAT homodimers in the absence of STAT1-STAT2-IRF9 heterotrimers (Fig. 4*F*). Thus, IP-10 induction in response to stimulation with 1 ng/mL type IFN of any of the three subtypes was severely or totally abolished in the whole-blood samples of patients with genetic defects of the type IFN pathway tested. As in patients with auto-Abs neutralizing type I IFNs, these inborn errors of the type I IFN response pathway impaired type I IFN-induced IP-10 production. These findings suggest that this assay can be used to detect both genetic defects of the type I IFN response pathway (as shown for mutations of *IFNAR1*, *IFNAR2*, *TYK2, IRF9*, and possibly *STAT1*, *STAT2*, and mutations of *STAT1* also affecting the type II IFN pathway) and auto-Abs neutralizing type I IFNs.

## Discussion

Human IP-10 was initially reported to be strongly induced by type II IFN in U937 cells (97). It is also induced by type I and III IFNs (also known as IFN-λs: IFN-λ1 (IL-29), IFN-λ2 (IL-28a), IFN-λ3 (IL-28b), and IFN-λ4) in human umbilical vein endothelial (HUVEC) cells and is, therefore, considered to be an ISG without selectivity for any particular type of IFN (98–100). The function of human IP-10 at whole-body level is unknown, as no IP-10-deficient humans have ever been reported. Mice with genetic defects resulting in an absence of IP-10 display impaired T-cell proliferation in response to allogeneic and antigenic stimulation, impaired IFN-γ secretion by T cells in response to antigenic challenge, and an impaired ability to control the replication of mouse hepatitis virus in the brain (97). We show here that human IP-10 is induced by IFN-α2, IFN-β, IFN-ω, or IFN-γ, as shown by LEGENDplex™ and ELISA, in whole blood from healthy individuals, but not in whole blood from patients with circulating auto-Abs neutralizing the corresponding type I IFNs, or patients with inborn errors of immunity affecting the type I IFN response pathway. The detection of IP-10 by ELISA or ELISA-like techniques is, therefore, a suitable readout when screening for common auto-Abs neutralizing type I IFNs and, possibly, rarer auto-Abs neutralizing type II IFN. The prior incubation of blood from healthy donors with mAbs that block IFNAR1 or IFNAR2 can be used as a control. Human IFN-γ can be used as a control in assays screening for auto-Abs neutralizing type I IFNs or genetic defects of the type I IFN receptor. High concentrations of type I IFNs could also be used as a control, although this would increase the cost of the assay, and an appropriate concentration of IFNs not neutralized by potent auto-Abs remains to be determined. The use of whole blood rather than plasma or serum in this assay indirectly allows the detection, or at least the suspicion of auto-Abs neutralizing any form of human type I IFN or genetic defects of the type I IFN response pathway. Indeed, there is no other known mechanism that could explain a lack of response to IFN-α2, -β, and/or -ω in a patient whose leukocytes respond to IFN-γ.

This assay has several advantages over the classic neutralization assays performed in research laboratories (26). First, it is based on whole-blood stimulation and does not, therefore, require the prior processing of blood samples with the purification of cells, plasma, or serum. Second, the stimulation step is both easy and rapid (6 h overnight), providing a convenient organization of testing over a period of less than 2 d that is compatible with standard work schedules in diagnostic laboratories. Third, the plasma samples collected and left unstimulated (NS conditions) can be analyzed directly by ELISA for the structural detection of auto-Abs to corroborate any neutralization observed. Fourth, various types of ELISA are commonly used in diagnostic laboratories, as these assays are cheap and robust, with a high throughput, and can easily be scaled up. Fifth, the assay provides results rapidly (potentially within 10 h from the start of the assay, taking into account the times required for stimulation and IP-10 detection), and certainly more rapidly than the classical assays used in research laboratories (2 to 4 d). Sixth, the materials and reagents required are already in use in most, if not all diagnostic laboratories. No sophisticated or expensive machines are needed. Seventh, the cost of this assay is reasonable, at an estimated US$3 to 5 per sample and set of conditions, about half the cost of previous tests. Tubes precoated with IFNs could be used for blood-sample collection, making it possible to stimulate extremely fresh blood and, thus, to obtain an optimal IP-10 response. Overall, this assay is a sensitive and robust tool for the detection of auto-Abs neutralizing IFNs and inborn errors of the type I IFN response pathway, and is compatible with the technical constraints of a diagnostic laboratory. This is important clinically, given that auto-Abs neutralizing type I IFNs are not only predicted to occur in at least 100 million people worldwide, but have already been shown to underlie various life-threatening viral diseases, including viral pneumonias and encephalitis (10, 15, 26, 37, 71, 75, 101, 102).

## Methods

### Whole-Blood Stimulation with IFNs.

Fresh blood from healthy donors, IFNAR2- or TYK2-deficient patients, or individuals with auto-Abs neutralizing type I IFNs was collected in lithium heparin tubes (except for the comparison between lithium heparin, EDTA, and citrate tubes). The healthy donors were blood donors recruited through the French Blood Bank, aged 18 to 68 y and with no known medical or genetic condition. The blood was stimulated with a serial 10-fold dilution of human glycosylated IFN-α2 (Merck, Catalog No.: H6041-10UG), IFN-β (Peprotech, Catalog No.: 300-02BC), IFN-ω (Origene, Catalog No.: TP721113), IFN-ε (Biotechne, Catalog No.: 9667-ME-025), IFN-κ (Cusabio, Catalog No.: CSB-YP889172HU), IFN-γ (IMUKIN) mouse IFN-α2 (R&D, Catalog No.: 12100-1) or cynomolgus monkey IFN-α2 (R&D, Catalog No.: 14110-1), or was left unstimulated (NS), in a final volume of 200 or 500 µL. After 16 h (or less in time-course experiments) of stimulation at 37 °C, under an atmosphere containing 5% CO_2_, the plasma was collected and stored at −80 °C or used directly for the assessment of cytokine or chemokine production. The mAbs used as controls to block type I IFNs were preincubated with blood and used at a concentration of 3 µg/mL (anti-IFNAR2 mAb, PBL, Catalog No.: 21385) or at a dilution of 1:50 (Human Type I IFN Neutralizing Antibody Mixture, PBL, Catalog No.: 39000). A total minimal volume of 1 mL was sufficient for the testing of a blood sample in all the above conditions. The healthy donors tested were aged 20 to 69 y old, and this group included both men and women.

### Recruitment and Ethics.

The healthy donors were deidentified. Biological samples from IFNAR2- or TYK2-deficient patients were obtained from the referring clinicians, upon verification that a signed consent was available for each participant in the study. All the experiments involving human subjects conducted in this study were performed in accordance with institutional, local, and national ethical guidelines. Approval was obtained from the French Ethics Committee “Comité de Protection des Personnes,” the French National Agency for Medicine and Health Product Safety, the “Institut National de la Santé et de la Recherche Médicale,” in France (Protocol No. C10-16, ID-RCB No. 2010-A00650-39), and the Rockefeller University Institutional Review Board in New York (Protocol No. JCA-0699).

### Assessment of the Type I IFN Response in Whole Blood.

#### LEGENDplex™.

The type I IFN response was assessed with the LEGENDplex™ Human Anti-Virus Response Panel (13-plex) (Biolegend, Catalog No.: 740349), the LEGENDplex™ Human Inflammation Panel 1 (13-plex) (Biolegend, Catalog No.: 740809), and the LEGENDplex™ HU Proinflammatory Chemokine Panel 1 (13-plex) (Biolegend, Catalog No.: 740985) according to the manufacturer’s protocol.

#### IP-10 ELISA.

IP-10 detection was performed by ELISA on the plasma supernatant after whole-blood stimulation with IFN, in accordance with the manufacturer’s protocol (R&D, Catalog No.: DIP100).

#### CD169 ELISA.

CD169 detection was performed by ELISA on the plasma supernatant after whole-blood stimulation with IFN, in accordance with the manufacturer’s protocol (Abcam, Catalog No.: ab213757).

#### RNAseq.

Two million PBMCs were obtained from each of three healthy donors and stimulated with glycosylated IFN-α2 (Merck, Catalog No.: H6041-10UG) for 6 h in Roswell Park Memorial Institute (RPMI) medium + 10% FBS. RNA was then extracted with an RNA extraction kit (Zymo Research, Catalog No.: ZR1051). Total RNA sequencing was performed with an Illumina NovaSeq S2 flow cell (read length: 100 bp) with a read depth of 30 M. All FASTQ sequences passed quality control tests and were aligned with the GRCh38 reference genome with STAR (2.6.1d). BAM files were converted to a raw-count expression matrix with featurecount. Raw-count data were normalized with DEseq2. The ensemble ID targeting multiple genes was collapsed (average) and a final gene data matrix was used for a modular repertoire analysis, as previously described (103) or for geneset enrichment analysis (GSEA: fgsea) with hallmark gene sets (http://www.gsea-msigdb.org/).

#### scRNAseq.

For scRNASeq analysis following stimulation, we isolated fresh PBMCs from three healthy donors, and washed them three times with PBS plus 0.5% FCS. We fixed the cells with the Evercode^TM^ Cell Fixation v2 kit (Parse Biosciences, Catalog No. ECF2101), according to the manufacturer’s protocol. We then prepared libraries with the Evercode^TM^ WT v2 kit (Parse Biosciences, Catalog No.: ECW02135) and sequenced them with an Illumina NovaSeq 6000 sequencer. We sequenced about 10,000 cells per sample. scRNA-seq FASTQ files from 8 sublibraries were demultiplexed into 22 samples with the splitpipe pipeline (v1.0.4p) from Parse Biosciences. Demultiplexed FASTQ files from HD1, HD2, and HD3 were aligned with the GRCh38/hg38 human reference genome with standard alignment protocols. The expression matrices were integrated with the Seurat package (v4.3.0) in R. Initial quality control was performed manually based on standard metrics to filter out low-quality data. The filtered data were further integrated with Harmony(104). Cell-type annotation involved two sequential rounds of graph-based clustering. Clusters were identified on the basis of canonical marker gene expression, facilitated by the SingleR pipeline (105) with reference to the MonacoImmuneData (106). Pseudobulk differential expression analysis was performed with DESeq2 (103). The results were visualized with volcano plots generated with the EnhancedVolcano package (107) in R.

#### RT-qPCR.

RNA was isolated from peripheral blood mononuclear cells with the Zymo Quick-RNA Miniprep Kit (Zymo Research, Catalog No.: ZR1051), according to the manufacturer’s protocol. Reverse transcription was performed with the High-Capacity RNA-to-cDNA™ Kit (Applied Biosystems, Catalog No.: 4368814), according to the manufacturer’s protocol. The cDNA obtained was then subjected to qPCR with Applied Biosystems Taqman assays for CXCL10 and the β-glucuronidase (GUS) housekeeping gene for normalization, and with Fast Advanced Master Mix - Taqman™ Real-Time PCR (Applied Biosystems, Catalog No.: 4444557). The results are expressed according to the ΔCt or ΔΔCt method.

### Statistical Analysis.

For each set of stimulation conditions, the distributions of IP-10 quantification data in pg/mL were compared between the HD and APS-1 groups in nonparametric Mann–Whitney tests implemented in GraphPad Prism.

## Supplementary Material

Appendix 01 (PDF)

## Data Availability

All study data are included in the article and/or *SI Appendix*.
