# Supplementary material for: A sensitive assay for measuring whole-blood responses to type I IFNs
Source: Proc Natl Acad Sci U S A. 2024 Sep 23;121(40):e2402983121. doi: 10.1073/pnas.2402983121 (PMC11459193; doi:10.1073/pnas.2402983121)
Supplement: Supplementary file 1 — Appendix 01 (PDF) [file pnas.2402983121.sapp.pdf]

PNAS

Supplementary Information for

A sensitive assay for measuring whole-blood responses to type I IFNs

Adrian Gervais, Corentin Le Floc'h, Tom Le Voyer, Lucy Bizien, Jonathan Bohlen, Fatih Celmeli, Fahd Al-Qureshah, Cécile Masson, Jérémie Rosain, Marwa Chbihi, Romain Lévy, Riccardo Castagnoli, Anya Rothenbuhler, Emmanuelle Jouanguy, Qian Zhang, Shen-Ying Zhang, Vivien Béziat, Jacinta Bustamante, Anne Puel[*], Paul Bastard[*], Jean-Laurent Casanova[*,@]

[*] Equal contributions

[@] Correspondence: casanova@rockefeller.edu

This PDF file includes:

Figures S1 to S2

Supplemental Figure 1

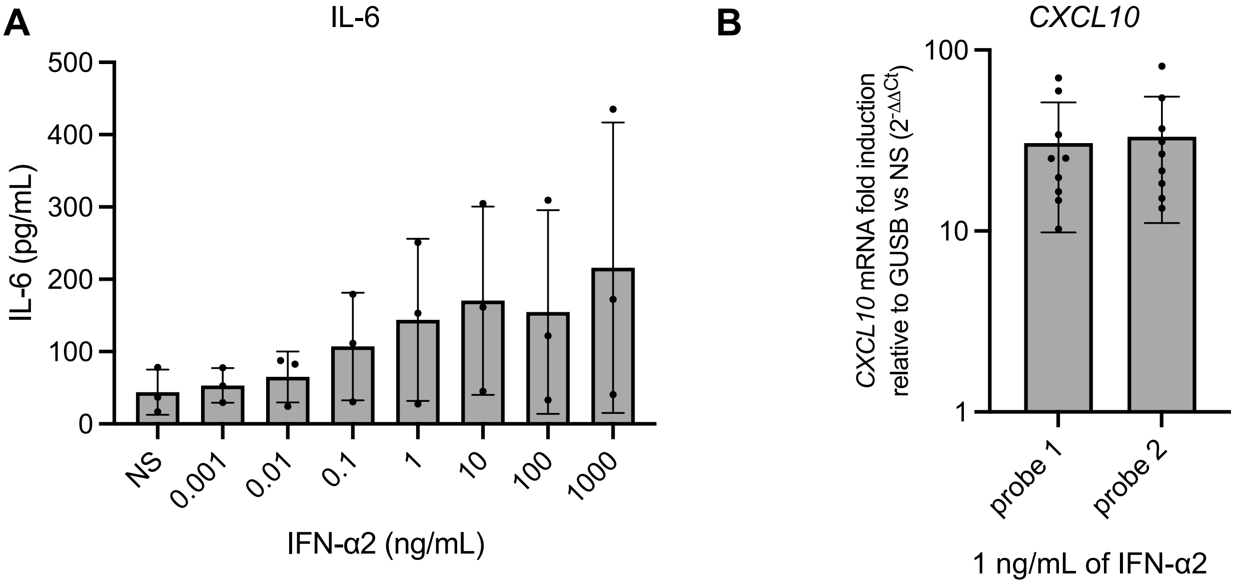

Supplemental Figure 2

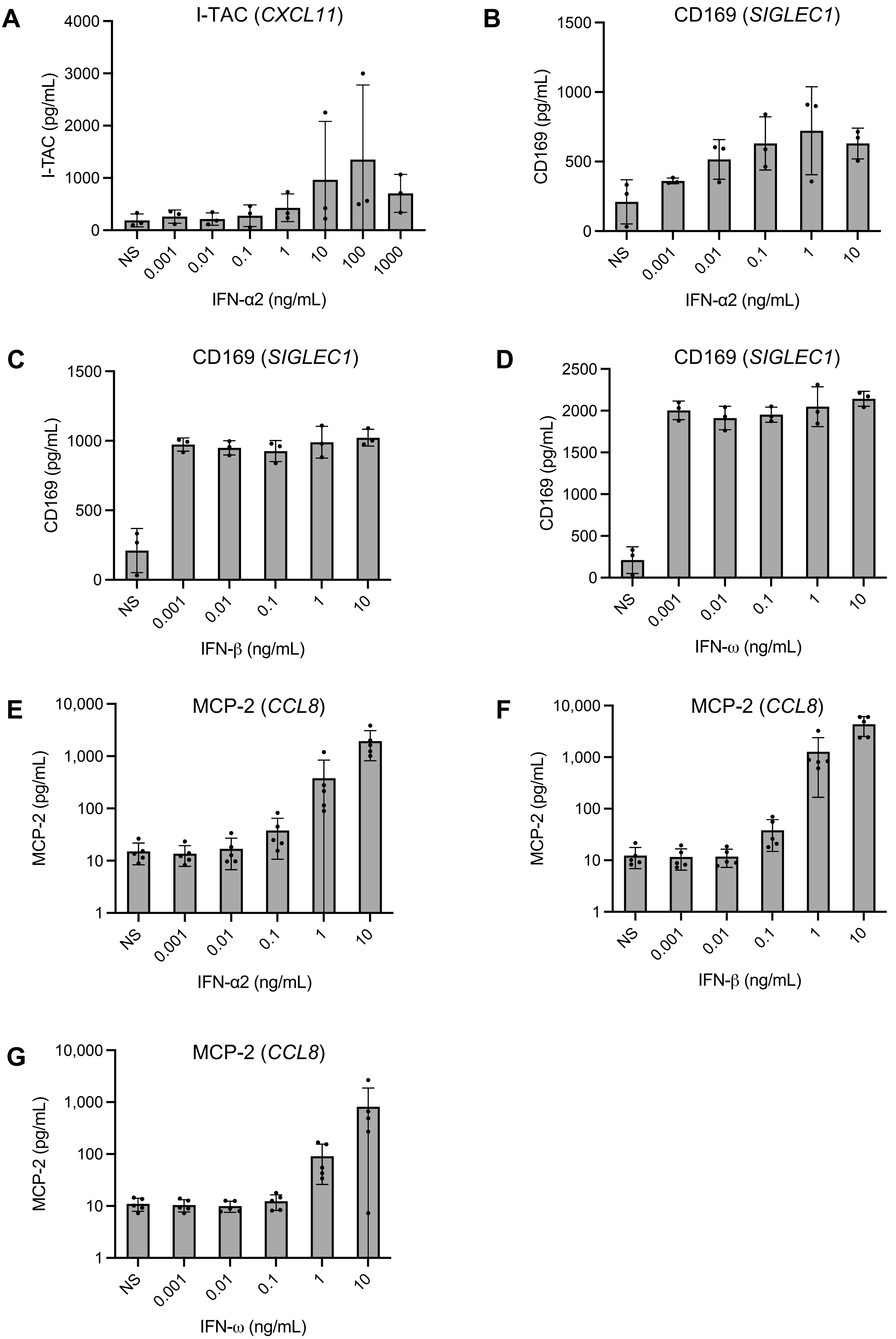

Supplementary Figures

Supplemental figure 1

(A) IL-6 induction after the stimulation of whole blood from three healthy donors with various concentrations of glycosylated IFN-α2 for 16 h. IL-6 levels were assessed in plasma supernatants by LEGENDplex™. **(B)** *CXCL10* fold-induction after the stimulation of PBMCs from nine healthy donors with 1 ng/mL glycosylated IFN-α2 for 6 h, as assessed by RT-qPCR.

Supplemental figure 2

Assessment of other potential target proteins after the stimulation of whole blood from healthy donors with type I IFNs (A) I-TAC induction after the stimulation of whole blood from three healthy donors with various concentrations of glycosylated IFN-α2 for 16 h. I-TAC levels were assessed in plasma supernatants by LEGENDplex™. **(B-D)** CD169 induction after the stimulation of whole blood from three healthy donors with glycosylated IFN-α2, IFN-β or IFN-ω. CD169 levels were assessed by ELISA. **(E-G)** MCP-2 induction after the stimulation of whole blood from three healthy donors with various concentrations of glycosylated IFN-α2, IFN-β and IFN-ω. MCP-2 levels were assessed in plasma supernatants by LEGENDplex™.
